# Supplementary material for: Restoration of Vegetation Greenness and Possible Changes in Mature Forest Communities in Two Forests Damaged by the Vaia Storm in Northern Italy
Source: Plants (Basel). 2023 Mar 19;12(6):1369. doi: 10.3390/plants12061369 (PMC10059996; doi:10.3390/plants12061369)
Supplement: Supplementary file 1 [file plants-12-01369-s001.zip › Table S1.pdf]

Table S1 Syntaxonomic scheme and other quoted syntaxa

**VACCINIO-PICEETEA Br.-Bl. in Br.-Bl. et al. 1939**
PICEETALIA EXCELSAE Pawłowski et al. 1928
Piceion excelsae Pawłowski et al. 1928
Vaccinio-Abietenion Oberd. 1962
Calamagrostio arundinaceae-Piceetum Andreis, Armiraglio, Caccianiga & Cerabolini 2009
Calamagrostio villosae-Abietetum Ellenberg et Klötzli 1974
**ROBINIETEA Jurko ex Hadac et Sofron 1980**
SAMBUCETALIA RACEMOSAE Oberd. ex Doing 1962
Sambuco-Salicion capreae Tüxen et Neumann ex Oberdorfer 1957
Piceo abietis-Sorbetum aucupariae Oberdorfer 1978
Rubetum idaei Kaiser 1926
**CRATAEGO-PRUNETEA Tx. 1962**
PRUNETALIA SPINOSAE Tx. 1952
Astrantio-Corylion avellanae Passarge 1978
Other quoted phytosociological classes (in alphabetical order):<br>• Artemisietea vulgaris Lohmeyer et al. in Tx. ex von Rochow 1951<br>• Asplenietea trichomanis (Br.-Bl. in Meier et Br.-Bl. 1934) Oberd. 1977<br>• Betulo carpaticae-Alnetea viridis Rejmànek ex Bœuf, Theurillat, Willner, Mucina et Simler in Boeuf et al. 2014<br>• Carpino-Fagetea sylvaticae Jakucs ex Passarge 1968<br>• Epilobietea angustifolii Tx. et Preising ex von Rochow 1951<br>• Erico-Pinetea Horvat 1959<br>• Festuco-Brometea Br.-Bl. et Tx. ex Soó 1947<br>• Loiseleurio procumbentis-Vaccinietea Eggler ex Schubert 1960<br>• Molinio-Arrhenatheretea Tx. 1937<br>• Montio-Cardaminetea Br.-Bl. et Tx. ex Klika et Hadač 1944<br>• Mulgedio-Aconitetea Hadac et Klika in Klika et Hadac 1944<br>• Nardetea strictae Rivas Goday et Borja Carbonell in Rivas Goday et Mayor Lòpez 1966<br>• Quercetea robori-petraeae Br.-Bl. et Tx. ex Oberd. 1957<br>• Trifolio-Geranietea sanguinei T. Müller 1962
